# Toward a human‐centric co‐design methodology for AI detection of differences between planned and delivered dose in radiotherapy

**DOI:** 10.1002/acm2.70071

**Published:** 2025-03-31

**Authors:** Luca M. Heising, Frank Verhaegen, Stefan G. Scheib, Maria J. G. Jacobs, Carol X. J. Ou, Viola Mottarella, Yin‐Ho Chong, Mariangela Zamburlini, Sebastiaan M. J. J. G. Nijsten, Ans Swinnen, Michel Öllers, Cecile J. A. Wolfs

**Affiliations:** ^1^ Department of Radiation Oncology (Maastro), GROW Research Institute for Oncology and Reproduction Maastricht University Medical Centre+ Maastricht The Netherlands; ^2^ Department of Management, Tilburg School of Economics and Management Tilburg University Tilburg The Netherlands; ^3^ Varian, a Siemens Healthineers Company Baden‐Dättwil Switzerland

**Keywords:** artificial intelligence, dose‐guided radiotherapy, explainable artificial intelligence, human‐AI interaction, human‐centric design, in vivo dosimetry, radiotherapy

## Abstract

**Introduction:**

Many artificial intelligence (AI) solutions have been proposed to enhance the radiotherapy (RT) workflow, but limited applications have been implemented to date, suggesting an implementation gap. One contributing factor to this gap is a misalignment between AI systems and their users. To address the AI implementation gap, we propose a human‐centric methodology, novel in RT, for an interface design of an AI‐driven RT treatment error detection system.

**Methods:**

A 5‐day design sprint was set up with a multi‐disciplinary team of clinical and research staff and a commercial company. In the design sprint, an interface was prototyped to aid medical physicists in catching treatment errors during daily treatment fractions using dose‐guided RT (DGRT) with a portal imager.

**Results:**

The design sprint resulted in a simulated prototype of an interface supported by all stakeholders. Important features of an interface include the AI certainty metric, explainable AI features, feedback options, and decision aid. The prototype was well‐received by expert users.

**Conclusion/discussion:**

Using a co‐creation strategy, which is a novel approach in RT, we were able to prototype a novel human‐interpretable interface to detect RT treatment errors and aid the DGRT workflow. Users showed confidence that the overall design method and the proposed prototype could lead to a viable clinical implementation.

## 1 INTRODUCTION

**FIGURE 1:**
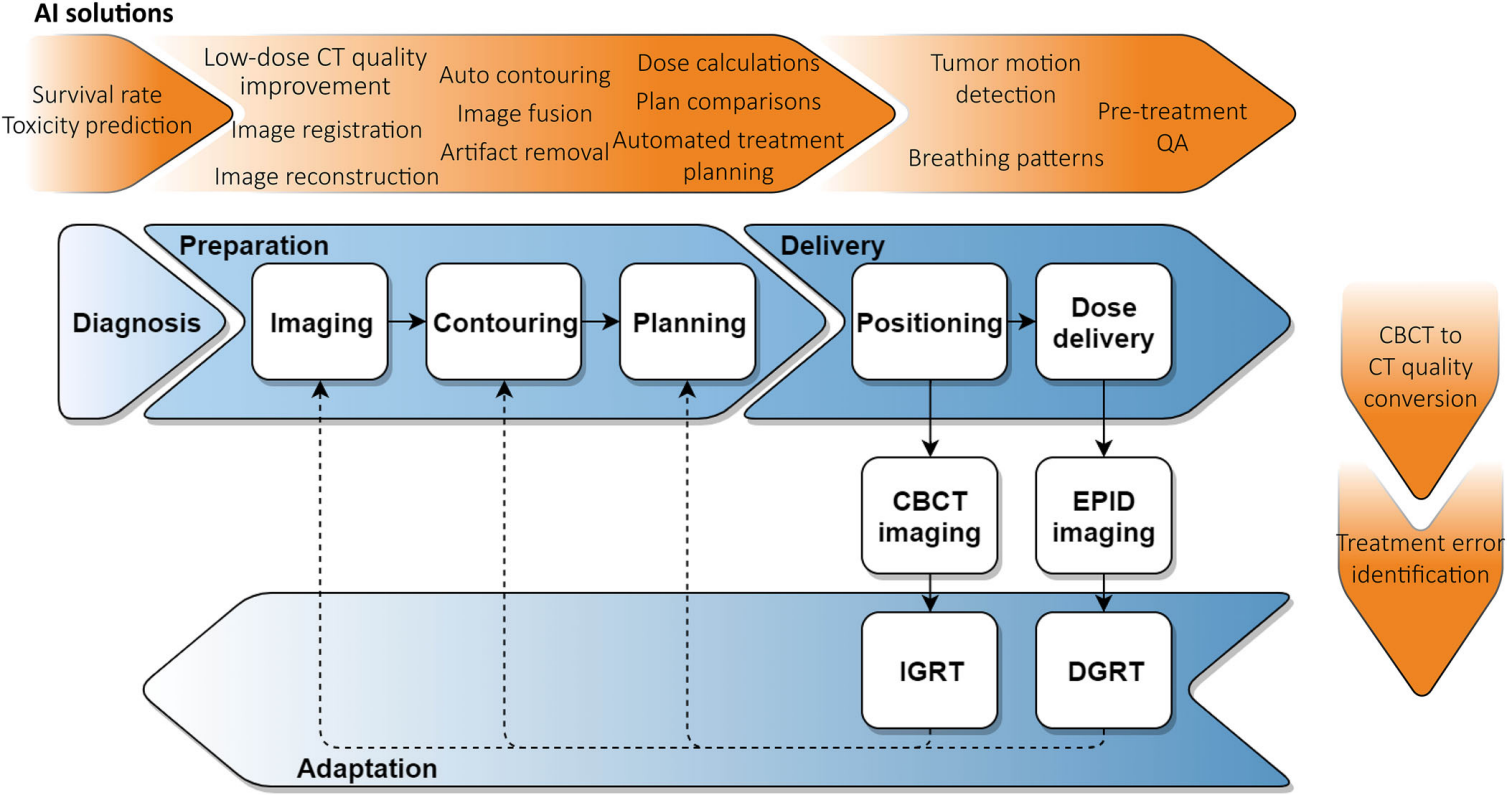
The radiotherapy workflow including AI applications proposed (in orange) to aid the steps of the workflow (adapted from Wolfs^8^).

Emerging research is exploring the enhancement of various steps in the radiotherapy (RT) workflow through artificial intelligence (AI) (Figure 1). This would enable greater automation and streamlining of the workflow, allowing clinical staff to dedicate more time to complex cases and deliver improved, personalized care. In the current RT landscape, a challenging AI implementation gap has appeared,^1^, ^2^ namely, the gap between the development and implementation of AI. While AI promises to transform RT, enhancing the efficiency and effectiveness of RT treatment,^3^, ^4^ the number of currently widely implemented AI solutions into RT is limited to AI‐driven auto‐contouring and to a lesser extent auto‐planning. The AI implementation gap has become one of the major issues for advancing AI in RT. Recent work illustrates that researchers and clinicians are frequently misaligned regarding expectations and requirements related to AI and its interpretation.^5^, ^6^, ^7^ Specifically, the work by Huet‐Dastarac et al. shows how clinicians prefer different visualizations for segmentation uncertainty than the researchers initially thought.^6^ According to Cabitza et al., this gap is a challenge that requires a solution from various perspectives. One of their suggestions highly focuses on the human aspect related to trust, availability, and usability.^2^

Addressing the AI implementation gap requires bridging the misalignment between AI researchers and AI.^7^ Many AI researchers propose great solutions without addressing what is deemed a problem by clinicians: “A hammer in search of a nail.”^9^ The COVID period reflects this phenomenon as the problem amplified rapidly. In a very short time, numerous AI algorithms were published for first‐line diagnosis of COVID‐19 on radiography images. Although the AI applications achieved great performance, none were applied in clinical practice because the AI did not offer considerable benefits compared to the conventional methods.^9^ This raises the question of how AI systems could contribute to better patient care in practice, and how AI can best be designed as an actionable aid for clinical users.

The challenge of translating clinical research into practice is not exclusive to AI. This phenomenon is observed with other technological and non‐technological innovations as well.^10^ However, AI introduces additional complexities due to the inherent opaqueness resulting in a lack of interpretability and understandability.^11^ AI algorithms differ fundamentally from conventional types of technology used in RT due to their self‐learning mechanisms. As a result, AI operates as a black box, uninterpretable to its users, creating a barrier between the users and the AI system.^12^ AI opacity was found to be the most important obstacle to AI implementation.^13^

This emphasizes the need for a human‐centered AI interface design, especially in medicine, where patient safety is at stake and lack of trust towards AI is a big issue. Human‐centered design is a technique that centralizes the needs and demands of users and other stakeholders in the development process instead of the technical capabilities of the AI. This technique advocates an intuitive and easy‐to‐learn design for end users required for effective human‐AI collaboration.^12^

Many studies have emphasized the need for human‐centered AI design, particularly in the context of human‐AI interaction.^12^, ^14^, ^15^, ^16^, ^17^, ^18^, ^19^ However, the number of publications in RT that adopt a human‐centered approach remains limited. This approach can be challenging because it differs significantly from the traditional innovation development processes familiar to the field of RT. In this study, we present a human‐centric approach to designing human‐AI interactions.

To exploit AI to its fullest potential, we aim to address the AI implementation gap by proposing a human‐centric design for a user‐friendly interface of an AI‐driven RT treatment error detection system. This study focuses on introducing the co‐design sprint methodology to RT, showcasing the design process and the developed interface for an in vivo dosimetry (IVD) use case as a tool for adaptive radiotherapy (ART).

## 2 METHODS

### 2.1 Use case

ART offers an opportunity for improved RT care driven by AI. With ART, the RT treatment plan is adjusted based on detected anatomical or other changes, aiming to enhance treatment accuracy by performing corrections.^20^, ^21^ Proposed solutions to effectively perform ART on a large scale rely on fast AI, as manual error detection and adaptation for every patient would be unfeasible. Nevertheless, in practice, these AI tools are rarely implemented.

IVD aims to catch treatment errors, monitor the actual dose delivered to the patient, and assist in treatment adaptation (ART).^22^ For external beam radiotherapy (EBRT), the electronic portal imaging device (EPID) lends itself well for IVD and presents the advantage of passively measuring dose without needing further setup. In practice, IVD using EPID is rarely performed as assessing the complex multitude of EPID‐generated information is too time‐consuming to act upon treatment errors in time, as required for dose‐guided (adaptive) radiotherapy (DGRT). Recent AI developments allow for rapid processing of these large quantities of complex EPID‐generated data to extract relevant information for further decision‐making.^21^ We have chosen IVD based on EPID data to showcase the design sprint methodology. In our use case, gamma maps (comparisons of reference and measured time‐integrated EPID images) are used as input data to the AI algorithm to classify errors that may occur during RT.^23^ This use case has faced obstacles in progressing toward implementation since the EPID data and the AI solution are intangible to target users.

To promote IVD for clinical practice, this study focuses on the design of an interface, named DGRT.AI, to facilitate treatment verification and treatment error detection by analyzing EPID images using AI, while presenting human‐interpretable information to users. It should be noted that EPID dosimetry is used as an example here to introduce the human‐AI co‐design process in RT. However, the process is sufficiently general and can be applied to a large range of clinical tasks that can be automated, and that require interaction with clinical staff, including other types of treatment error detection.

### 2.2 Human‐AI co‐design strategy

For the design of our AI‐driven DGRT solution, we introduce a human‐centric design approach based on design thinking.^24^ Key principles of human‐centric design include (1) leveraging users’ experiences, knowledge, and behavioral patterns, (2) adapting the system to users’ familiar work practices, and (3) minimizing cognitive load and interruptions.^14^ Cognitive overload is especially relevant in medical systems, as it can contribute to fatigue, stress, and impaired decision‐making, potentially leading to harmful outcomes.^25^

Commonly, in RT, a design process stays at the research stage until the product is presented to the intended end‐users. This may lead to clinical non‐acceptance and requests for major re‐design efforts. Making subsequent changes can prolong the process, and sometimes, large changes become infeasible. RT innovations typically follow the technology readiness level (TRL) ladder to determine the *readiness* to get innovations to the next stage.^26^ In this type of common approach, many stages may have passed before the innovation is presented to the real world where researchers and engineering personnel learn from the experience of end‐users. Employing “Design Thinking,” and more specifically a design sprint, speeds up the learning process and aligns the needs of clinical practitioners with design characteristics of the AI tool.

**FIGURE 2:**
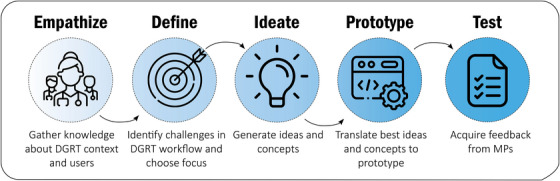
Five phases of the Design Thinking process for the proposed AI‐driven in vivo dosimetry application. This process was concluded in 5 consecutive days. DGRT, dose‐guided radiotherapy; MP, medical physicists.

Design Thinking is a research‐based innovation process that prioritizes end‐users’ needs and challenges, instead of first progressing through a lengthy development process before presenting a finished product to end‐users.^24^ This approach promises greater implementation success. Furthermore, Design Thinking is an analytical and creative process that involves engaging individuals in experimenting, creating prototypes, gathering feedback, and iteratively redesigning.^27^ In this study, a 5‐day design sprint was set up. Each day of the design sprint focused on one Design Thinking phase (Figure 2). With the proposed approach, end‐users are involved throughout the entirety of the research process, from the very first stage.

For the design of the DGRT‐AI prototype, experts were recruited to participate in the design sprint, from within our clinic and from a large commercial partner. In total, eight people participated in the design sprint. The team was a mix of academic researchers, clinical staff (physicists), and experts on either content or design from industry. The diversity of the design sprint resulted in healthy discussions and a good dynamic. Nevertheless, it should be noted that choosing the right people to participate might be a challenge. As is always the case when sampling, sampling bias may occur. To reduce this risk, we have tried to gather a diverse team from different professional backgrounds. Furthermore, besides the core design sprint team, six additional interviewees (four clinical and two industrial) were recruited for the *Empathize* and *Test* phase who were independent and participated only on the first and last day of the sprint to provide the team with additional insights. One decision‐maker was appointed to moderate discussions and finalize decisions during the sprint.

### 2.3 Phases of the Design Thinking process

#### 2.3.1 Phase 1: Empathize

Various stakeholders are involved in the ART process, such as RT oncologists, RT technologists, and medical physicists (MPs). MPs are primarily responsible for detecting treatment errors in the presented use case. Therefore, this design sprint focused on MPs as users of the DGRT.AI interface. The goal of the *Empathize* phase was to understand the MP's role and the challenges they face. First, the DGRT workflow was mapped followed by setting a collective goal for the product of the design sprint. The goal reflects the degree of complexity and innovativeness allowed for the prototype. To illustrate, if the goal is to launch a product in 5 years, there are more limitations than when the goal is set for 20 years. All team members of the design sprint proposed a goal after which a voting system was employed to gather opinions on the proposed goals. The decider chose one of the proposed goals and could swap out parts with parts from the other proposals. Two MPs and two quality assurance experts were interviewed to validate the DGRT workflow and identify challenges they faced in this workflow. The scope of our sprint was limited to supporting MPs in detecting treatment errors and their decision‐making related to DGRT. However, all challenges mentioned during the interviews were noted by the sprint team, even when the challenge was outside the scope of our sprint.

#### 2.3.2 Phase 2: Define

The *Define* phase directs the sprint team to reach the previously set goal. Identified challenges from the empathize phase that were related to one another were grouped into themes. Each theme had multiple challenges. Afterward, a voting system was used to select a single theme as the focus of the design sprint and prioritize the remaining challenges. Each member of the design sprint team got one vote for a theme and three votes for individual challenges. The latter could be freely chosen among all themes and challenges. This way, the team collectively prioritized the themes and challenges, guiding the decider in their decision. The decider chose one main theme to focus on during the design sprint, aligning with the overarching goal as determined in the *Empathize* phase.

#### 2.3.3 Phase 3: Ideate

In the *Ideate* phase, the sprint team drew low‐fidelity concepts for (elements of) the interface on paper (example of a low‐fidelity concept is provided in Appendix I) based on the chosen theme in *Define* and the overarching goal. Concepts could range from minor features to complete interfaces and very novel ideas. In the final step of the ideation phase, the participants presented their concepts. One concept was chosen using the aforementioned voting system to be prototyped.

#### 2.3.4 Phase 4: Prototype

A rapid prototype was developed using interface prototyping software Figma (Figma Inc, San Francisco, California, USA).^28^ The prototype was non‐functional but allowed for simulated interaction with users. Relevant images to assess detected treatment errors were simulated using in‐house software and the treatment planning system, including delineated computed tomography (CT) and cone beam CT (CBCT) images, EPID images, and dose volume histogram graphs. The representative images provided a realistic view of the envisioned AI interface. Furthermore, two storylines were drawn up for the presentation of the storyline; one storyline where the AI correctly identified a treatment error with high certainty and the other storyline where it misidentified an error with relatively low certainty.

#### 2.3.5 Phase 5: Test

Lastly, the prototype was tested with four end‐users (three MPs and one quality assurance expert). During interviews, the users were presented the prototype and both storylines and were asked to provide feedback on the prototyped interface. More specifically, they were asked what actions they would take and which information they would need in each scenario. Afterward, users were asked to rate the interface on a 7‐point Likert scale related to technology acceptance, to quantify how useful, easy to use, and trustworthy they perceived the system.^29^ Trust is a crucial factor for the integration of AI in clinical settings. Given its complexity, this study focuses specifically on trustworthiness, a key driver of trust. Trustworthiness can be defined as the extent to which the system meets its obligations or, in this context, the users' expectations.^30^

## 3 RESULTS

### 3.1 Empathize

The collectively defined goal of the DGRT.AI design sprint was: “In 5 years, with DGRT.AI based on EPID & AI, the MP will be able to confidently detect and verify treatment errors, including root cause analysis, quickly enough to support decision‐making, & taking actions when needed, always before the next fraction, to achieve safer treatment for every patient.”

Furthermore, 10 core challenges were identified associated with the DGRT workflow (Table 1).

**TABLE 1:** Themes emerging from the design sprint (Define phase)

Theme	Description	Example
Presentation of information	The right (detail of) information at the right time.	Able to see general information in one glance while able to dive into details when needed. Provide enough information for decision‐making without cognitive overload.
AI verification	Making the AI results verifiable and trustable.	Identifying misclassifications made by the AI.
Increase confidence	Confidence in the system.	How can the MP be confident that the error identified by the AI is correct?
Decision making	Aiding the decision‐making process.	What tools can support the MP in their decision‐making following the AI outcome?
Identifying trends	Development of errors over time.	An error that continuously exacerbates is more relevant than a randomly occurring error.
Reduce workload	Minimizing the (extra) workload.	Automating (part) of the workflow to minimize the time needed to evaluate all fractions.
Optimize workflow	Integration of the system in the RT workflow.	Not having to open different files and folders to be able to perform the dose verification.
Understanding of treatment errors	Defining clinical relevance of treatment errors.	A very small discrepancy in dose may not be relevant to the accuracy of the treatment.
Ideal preparation	Practical implications of EPID measurements.	Collision of EPID set up with other treatment preparation steps.
Measurement accuracy	EPID accuracy is vital for correct AI predictions.	If the EPID dosimetry is inaccurate, identifying accurate errors is unfeasible.

The first row represents the chosen theme for the focus of the design sprint. The first three rows represent themes with many votes. While not the primary focus, these themes were also taken into account during the design sprint. The lightest shaded rows were not actively targeted during the design sprint, but might be relevant for follow‐up sprints. Finally, the non‐shaded rows are challenges identified in the DGRT workflow but out of the scope of the DGRT.AI software development.

### 3.2 Define

After voting (Table 1), the sprint team decided to focus the prototype on the theme “Presentation of information.” Challenges grouped within this theme were, for example, “How to balance presenting required information with detailed information?”, and “How to present the right information at the right time?”. One of the challenges faced by the design team and experts was how to bridge a complex AI system and the end‐users, and how this complex information can be translated into easily digestible information, sufficiently informative for ART decision‐making.

### 3.3 Ideate

Many of the concepts proposed by the design team focused on the analysis of treatment errors and their presentation to support MPs, aligning with the sprint goal and the theme chosen in the *Define* phase. Proposed concepts included innovative ideas, such as a full 3D rendering of the delivered dose, or an AI voice assistant. In the chosen concept, the first screen of the system shows all relevant information to the user with the option to explore each treatment fraction further if desired. The main arguments for the chosen concept were the simplicity and ease of use. Other high‐scoring concepts presented more information on the first screen, such as graphs. Parts of other concepts that received many votes were included in the chosen concept. These were AI certainty estimations, an explainable AI (XAI) feature (e.g., indicating which regions of the EPID image were used to decide an error had occurred), and a decision aid based on pre‐defined protocols.

### 3.4 Prototype

The design sprint resulted in an interactive simulated prototype aligning with TRL 6. The most important features are outlined in the following section.

### 3.5 Presentation of information

**FIGURE 3:**
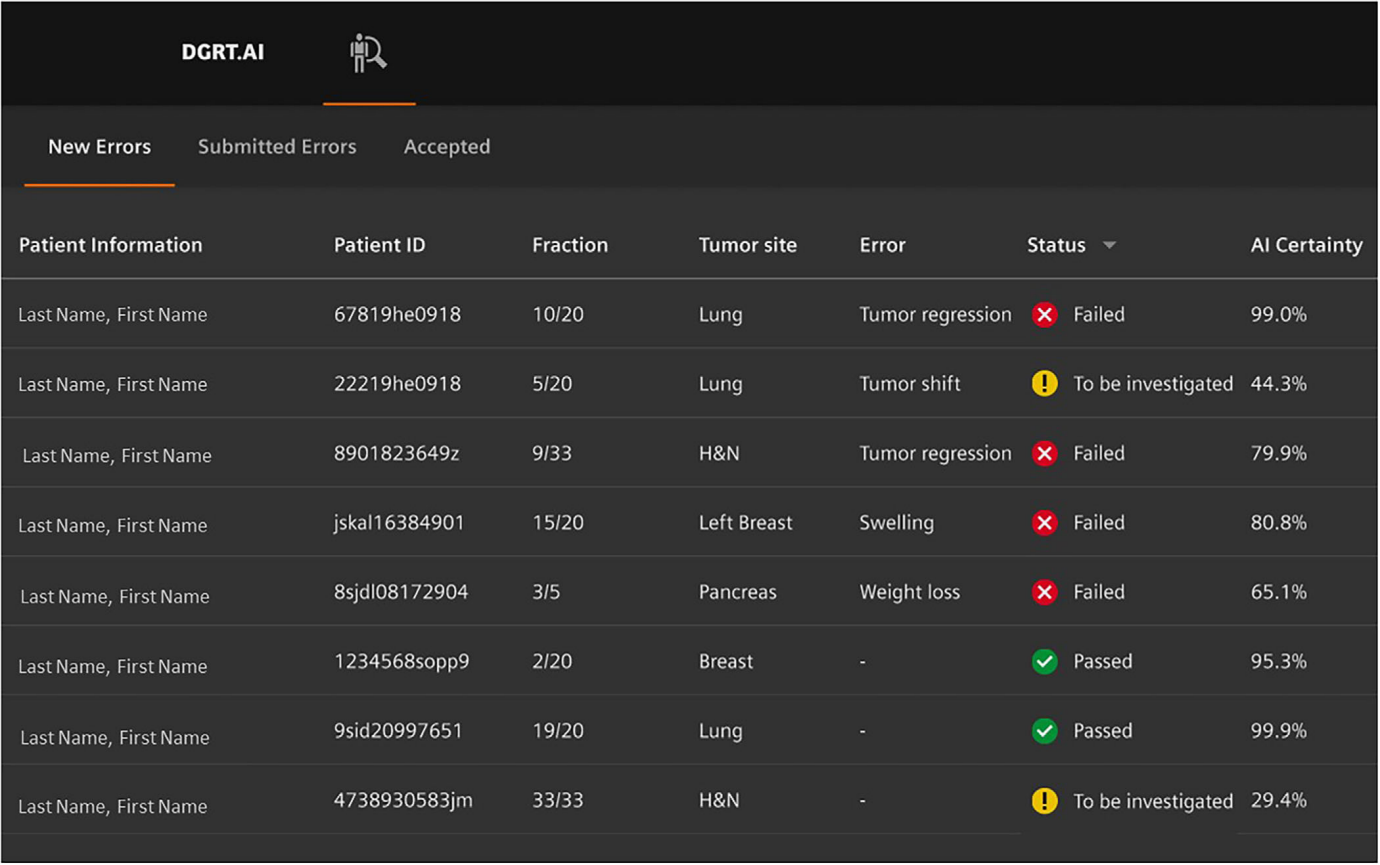
Snippet of the information presented in the top‐level overview. The interface shows the AI‐identified error. For an error with high certainty, the system will tell the MP that the fraction failed. In case of low AI certainty, manual investigation by the MP is required. NB: all information is fictitious.

In the first view of the interface, the user is presented a prioritized list of all treatment fractions for which IVD is performed. Relevant information about the patient is provided, such as name, patient ID, and current fraction number (Figure 3). The information provided in the first view is kept at a minimum with relevant information only. The AI‐based treatment error identification is provided as well, including whether the fraction passed or failed and how certain the AI is.

**FIGURE 4:**
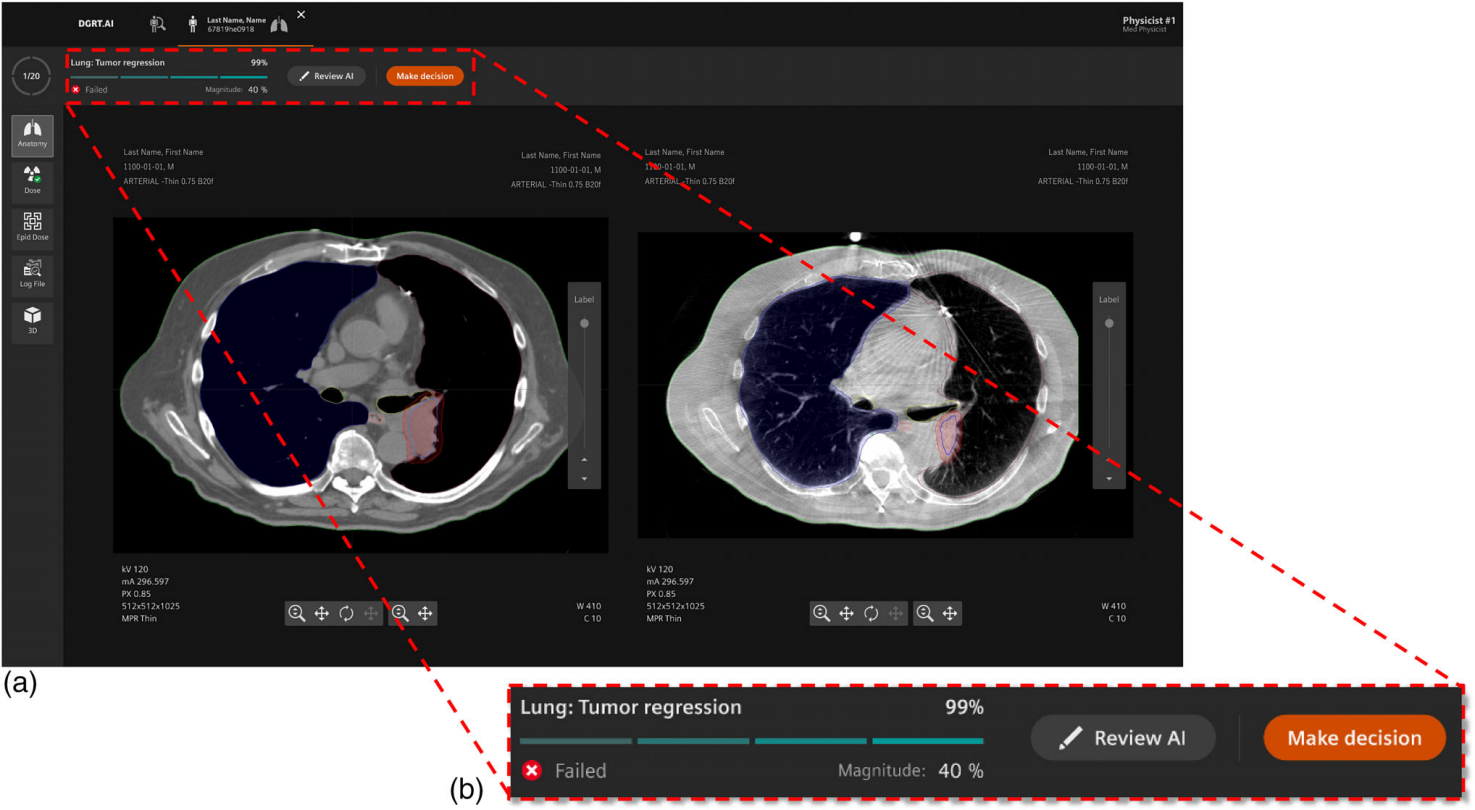
(a) Detailed view of a deeper level of the prototype showing the anatomical information of the patient. The medical physicist (MP) can choose another view (information about the anatomy, dose, EPID measurement, log files, or a 3D rendering of the dose) in the left ribbon. The upper red dashed area shows the AI‐classified error, including the certainty score, the magnitude of the error, and the options the MP has (Review AI or Make decision). (b) When the MP wants to make a decision, a pop‐up shows up with the best‐fitting follow‐up step to the detected error based on the embedded protocol. The “hospital most used” option indicates the most frequently chosen follow‐up steps for this error. NB: all information is fictitious.

By clicking on the fraction, information relevant to the AI‐classified treatment error is provided. In the example of tumor regression (Figure 4a), the most relevant information to confirm the AI error classification is anatomical information, so the planning CT and CBCT of the fraction are shown. If desired, the user can find additional information in the left ribbon, such as dose information, EPID images including gamma analysis, dose recalculation based on accelerator log files or EPID images, or a 3D rendering of the CT and dose.

### 3.6 AI treatment error detection

To identify treatment errors, a convolutional neural network trained on gamma maps was previously developed.^21^ This AI is able to detect ten different error types with 86.6% accuracy on synthetic data. Ongoing work aims to translate these results to real‐world data and to predict the error magnitude in addition to the error type. Applying this AI will thus result in an AI‐classified error type, which is shown in the first view of the prototype interface. When clicking on the fraction, the predicted error magnitude is visible in the upper left corner. The first view is automatically ordered by error occurrence so that the fractions containing an error are on top of the list.

### 3.7 AI certainty

An important property of the system is the AI certainty score (Figure 3). The AI certainty score informs users which fractions need to be manually assessed. The combination of the listed status (fraction pass/fail/to be investigated) and AI certainty informs users about the case complexity. Especially when the fraction passes without an error, but the AI certainty is low; users may choose to manually investigate the fraction considering it may be a false negative case, which is normally hard to detect. This might occur for smaller errors that are difficult to detect by the AI. Conversely, when an error is reported, but the AI has low certainty, the user may also wish to investigate deeper as this may be a false positive error, which may end up undergoing an unwarranted correction, potentially leading to an introduced error.

### 3.8 Decision‐making aid

In practice, the best decision following a treatment error can be unclear. In our design, we propose recommended follow‐up steps based on protocols embedded in the system (Figure 4b). We envision the embedded protocols to be developed based on predefined guidelines by the RT community (e.g., by ESTRO or AAPM), or clinics can add a local/institutional protocol. Another option is to view the hospital's most used decisions for the detected treatment error to evaluate which actions have been taken in similar cases (Figure 4b). Users can assign follow‐up steps to other users (e.g., RT oncologists or RT technicians) and include comments.

### 3.9 Explainable AI and feedback

If the user is skeptical about the AI prediction (e.g., due to low AI certainty), they can evaluate the AI explanation. In our prototype, a heatmap highlights areas of the input image that the AI considers most important for error detection. To avoid information overload to the user, the XAI information is available upon request. If the user agrees with the AI prediction after evaluating the XAI, they can accept the identified error and follow the same decision‐making steps as provided by the decision aid. If the user disagrees, they can provide feedback and correct the error type, which is saved in a database to continuously improve the AI model by regularly retraining the algorithm.

### 3.10 Test

During the test phase, the expert users noted the system's simplicity and the ease of use in their first reaction. Participating end‐users were asked how useful they perceived the simulated tool in their future daily work on a 7‐point Likert scale. Two users rated the tool as six (“agree”), and the other two users as seven (“strongly agree”). On the perceived ease of use, the system received evaluations between five (“somewhat agree”) and seven. When asked about perceived trustworthiness, the users were ambiguous. The users argued trustworthiness relates to experience with the system, including experimenting with some use cases, which is difficult to assess in the presented environment. The proposed system (as is) scored between a four (“neutral”) and a six on trustworthiness. Another feature that could increase the users’ trust is continuous improvement through user feedback. A more comprehensive overview of the end‐users’ feedback on the prototype can be found in Appendix III.

Some users had remarks related to the AI certainty estimation. While users acknowledged the added value of the certainty metric, they found it challenging to interpret the metric regarding when to investigate a fraction. Understanding how AI certainty is determined was noted as crucial for users to make informed decisions. Interestingly, all users immediately noticed the passed fraction with low certainty at the bottom of the list and expressed their intention to investigate this fraction even though the AI did not identify an error. In instances where no error was detected and AI certainty was high (e.g., 99%), end‐users expressed sufficient confidence to accept the fraction without manual investigation.

Users expressed appreciation for the ability to assign follow‐up tasks and add comments, facilitating smoother collaboration among different disciplines. The end‐users highlighted that decision‐making can be influenced by inter‐fraction error trends. In current practice, information of previous fractions is taken into account by MPs. For example, if a random error is detected early in the treatment, MPs may opt to monitor without adaptation. In the proposed prototype, functionality for trend analysis of such inter‐fraction errors can be included at a later stage.

The prototype visualized XAI as a heatmap overlaid over a gamma map. A gamma map evaluates a compressed dynamically delivered dose into a single 2D image and as such is difficult to interpret for users, including MPs. As a result, the XAI on top of the 2D gamma map was also found difficult to interpret. Proposed solutions to increase the interpretability included a combination of 3D dose information and anatomical information in the XAI to visualize the exact location of the flagged error.

## 4 DISCUSSION

Using a co‐design methodology, we were able to prototype a novel human‐interpretable interface to detect RT treatment errors based on AI and aid the DGRT workflow. Users showed confidence that the overall design method, novel for RT, and the proposed prototype could lead to a viable clinical implementation. Offering a simple intuitive design allowed to minimize the cognitive load, which was acknowledged by end‐users. This showcase of the process of Design Thinking in RT could be an example for a variety of AI development processes in the clinical field of RT.

The co‐design approach elicited promising reactions from end‐users. The prototype is tangible and concrete enough to learn from end‐users in a very early stage. At the same time, follow‐up steps need to be considered; this may include follow‐up design sprints. For example, some features in the prototype illustrated a concept that the expert users noted as highly desirable. To further design these concepts, shorter follow‐up design sprints could focus on just these features to further improve and refine. This was the case for the XAI and AI certainty features. Such features can be subject to another design sprint. In this way, the development process consists of subsequent sprints, building upon the knowledge gathered from preceding sprints.

### 4.1 Human‐centered AI‐driven interface design

The Design Thinking framework prioritizes end‐users in the design of an AI‐driven interface, with the purpose of communicating the complexity of AI comprehensively to end‐users. The end‐user can easily be overlooked in AI development since the development of AI inherently requires a lot of attention. During a design sprint, important factors to increase AI acceptance surface and can easily be incorporated into the design early on, such as important safety features, the degree of alert sensitivity, and characteristics of the stakeholders, for example, experience, background knowledge, and responsibilities.^31^ Based on previous work on success factors to innovations in RT, core characteristics of a successful (i.e., timely implementation) innovation are (1) competent and sufficient team members, (2) complexity, (3) understanding/awareness of the project goals and process by the team members, and (4) feasibility and desirability.^32^ The proposed user‐centric co‐design framework effectively guides the team in achieving these critical attributes. The Design Thinking framework acknowledges the four core characteristics of a successful innovation project. In the present study, we showcased how this framework can be applied in a design sprint method to achieve successful AI projects.

By incorporating key stakeholders, the method meets the objective of including competent and sufficient team members in the project. Since every team member contributed to the goal definition, all team members expressed they understood the project goals and took ownership of these goals. Furthermore, by concentrating on a single theme or challenge during the design sprint, the project's complexity was reduced effectively. More complex AI projects may be divided into multiple design sprints, further mitigating the overall complexity. Finally, the stakeholders (e.g., MPs, designers, researchers) expressed their concerns regarding feasibility and desirability to ensure these factors were considered from the project's start. The design‐sprint methodology presents a promising step to bridging the AI implementation gap to successfully implement proposed AI solutions to the RT workflow as presented in Figure 1.

Commonly noted bottlenecks in the implementation of AI in RT include a lack of knowledge and interpretation, trust and explainability issues, and challenges related to technical performance and workflow integration.^2^, ^33^, ^34^ Various studies suggest that human‐centric methods can effectively address these challenges.^14^, ^35^, ^36^, ^37^, ^38^ The method presented in this study demonstrates how such a human‐centric approach can be successfully realized.

### 4.2 Future work resulting from the design sprint

Some findings of the design sprint should be considered for further investigation. The AI certainty metric was important in MP's trust in the AI system. Computing a trustable AI certainty metric was not the focus of our prototype, but presents a challenge for further development, as there is no consensus on how to compute the overall uncertainty of an AI system. Elements that could be included are, for example, evaluating the role of the SoftMax probability,^39^ outlier detection, or Monte Carlo uncertainty.^40^ Hence, the development of an AI uncertainty metric in RT requires further research. The integration of the metric in the prototype could be a follow‐up to the design sprint presented here. The expert users emphasized the importance of understanding how this metric is computed to be able to interpret it accurately. This consideration is crucial for the research, development, and integration of a certainty metric.

Following the current study, we aim to investigate how the XAI feature should be designed to best fit the user's needs. XAI is a novel line of research that aims to explain why the AI outputs a certain prediction. The XAI requirements vary based on users’ background knowledge and needs. While MPs appreciated the XAI feature, the proposed solution was difficult to interpret. Follow‐up studies will focus on XAI tools that meet the requirements of a good explanation as defined by Buijsman,^41^ that is, an explanation that exhibits some form of causation. For example, by exploring counterfactual explanations or natural language explanations, which are two methods of achieving explanations that exhibit a form of causality as described by the ladder of causation.^42^, ^43^ Counterfactual explanation aims to discover the decision boundaries of the AI model. Natural language explanations aim to describe the AI decision in natural language so that qualities of good explanations can be included in the explanations.^41^ This way, the XAI can also be tailored to the end‐user.

One of the challenges identified during the *Empathize* phase was the time trend analysis of errors. Taking potential errors from previous fractions into account can emphasize the importance of a detected error and the urgency to investigate deeper. In ongoing work, we study how AI can detect systematic and random inter‐fraction treatment errors, which could be added into the AI interface in a later stage.

### 4.3 Challenges of the design sprint

Before employing a design sprint according to the Design Thinking principles, some general challenges should be considered. First, the design sprint requires active participation of end‐users throughout the entire process. It can be challenging to acquire dedicated time from staff in clinical settings due to the workloads they face. For this reason, positive use cases, such as presented in this study can contribute to showcasing the value of their invested time. Nevertheless, in some cases, it may be necessary to split up the sprint phases or speed up parts of the process. For follow‐up design sprints, it may be beneficial to include some of the same participants from the initial sprint. Although changing the design sprint team is feasible, the process might take longer if steps from previous sprints need to be repeated.

The final step of the design sprint should include a breakdown of actions to realize the deliverables of the end product. While the design sprint presents a promising first step to the development of the end product, the active involvement of end‐users should be considered throughout the entire development process. It is unclear what the exact impact of the design sprint is on the full process in terms of time savings and efficiency. What is clear is that the end‐users are imbued with a sense of ownership and continuous involvement, thereby helping to tailor the final product to their needs. Whether this can be sustained over the typically long timespan it takes from idea to final clinical product^44^, ^45^ remains to be seen. The presented use case was deemed appropriate for the design sprint methodology, which can be applied to other AI‐driven interfaces or applied clinical research, such as the design of clinical technology. Nevertheless, the methodology may not be the most appropriate for all RT studies. For example, for radical innovations, end‐users typically first show resistance toward the innovation,^46^ which can hinder the creative innovation development process. The design sprint could be used to build up toward radical innovation gradually.

## 5 CONCLUSION

A human‐centric co‐design approach, novel to the field of RT, was used to prototype an interface for automating AI for error detection. The system is designed to provide the user with sufficient information to evaluate each fraction at first glance and allows further exploration of the fraction and the certainty of the AI tool. The methodology engages the end‐users in the whole design process and instills ownership of the project. Novel components of the system include an AI certainty estimation, XAI and feedback component, and an AI‐driven decision aid.

## AUTHOR CONTRIBUTIONS

*Original writing*: Luca Heising. *Resources*: Viola Mottarella, Yin‐Ho Chong, Mariangela Zamburlini. *Conceptualization*: Luca Heising, Stefan Scheib, Viola Mottarella, Yin‐Ho Chong, Mariangela Zamburlini, Sebastiaan Nijsten, Ans Swinnen, Michel Öllers, Cecile Wolfs. *Supervision*: Frank Verhaegen, Maria Jacobs, Carol Ou, Cecile Wolfs. *Writing—Review & Editing*: Frank Verhaegen, Stefan Scheib, Maria Jacobs, Carol Ou, Cecile Wolfs. *Visualization*: Yin‐Ho Chong, Viola Mottarella, Mariangela Zamburlini, Cecile Wolfs. *Validation*: Ans Swinnen, Michel Öllers, Sebastiaan Nijsten.

## CONFLICT OF INTEREST STATEMENT

This study is part of a larger grant from Varian, a Siemens Healthineers company honored to Prof. Dr. Verhaegen. Additionally, Dr. Scheib, Dr. Zamburlini, Chong, and Mottarella are employed by Varian, a Siemens Healthineers company.

## Supporting information



Supporting information

Supporting information

Supporting information
