# Supplementary figures and images for: Toward a human‐centric co‐design methodology for AI detection of differences between planned and delivered dose in radiotherapy
Source: J Appl Clin Med Phys. 2025 Mar 31;26(6):e70071. doi: 10.1002/acm2.70071 (PMC12148753; doi:10.1002/acm2.70071)

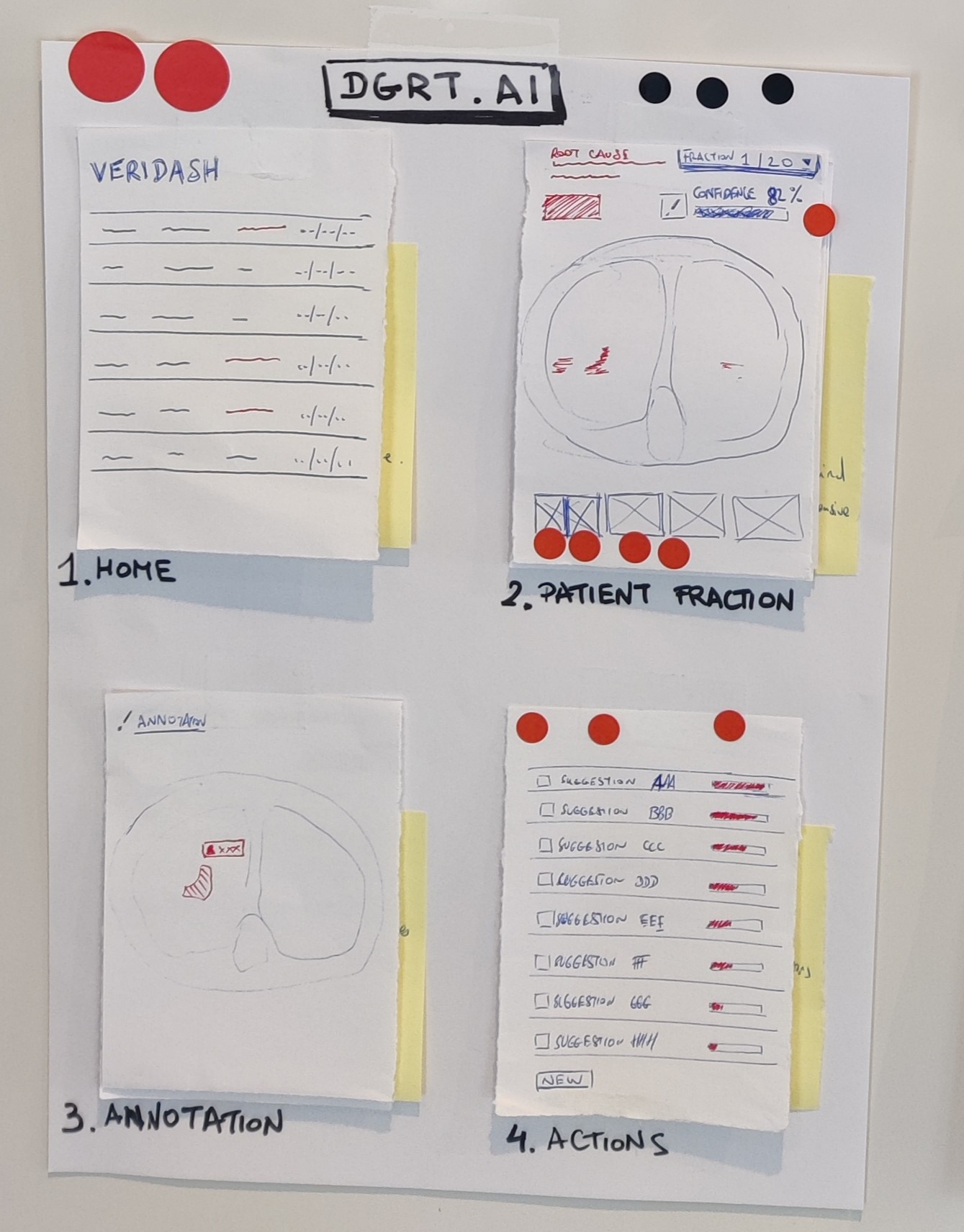

Supplement: Supplementary file 1 — Supporting information [file ACM2-26-e70071-s001.jpg]
