# Supplementary material for: Toward a human‐centric co‐design methodology for AI detection of differences between planned and delivered dose in radiotherapy
Source: J Appl Clin Med Phys. 2025 Mar 31;26(6):e70071. doi: 10.1002/acm2.70071 (PMC12148753; doi:10.1002/acm2.70071)
Supplement: Supplementary file 2 — Supporting information [file ACM2-26-e70071-s002.docx]

# Appendix I Low-fidelity concept

Example of a low-fidelity concept drawn on paper, including votes (red and black dots).

# Appendix II Generic planner

## Pre-sprint checklist

- **Define the scope of the challenge**

Sprints should be utilized when the stakes are high, time is limited, or when progress has stalled.

- **Decide on a Decider (or two)**

Without a Decider, decisions will not stick. The Decider must ensure the project's continuation post-sprint. If the Decider is unable to participate in the entire sprint, a delegate should be appointed to fulfill this role.

- **Recruit a sprint team**

Limit the team to seven members or fewer. Ensure the inclusion of diverse skills alongside those who work on the project daily.

- **Schedule extra experts**

An expert is an individual with knowledge pertinent to the challenge. This may include someone who wished to participate in the sprint but was unable to do so, someone with a deep understanding of the end-user, or an end-user themselves. For Monday afternoon, schedule three to five 30-minute interviews with additional experts. Schedule a total of three hours for these interviews.

- **Pick a Facilitator**

Choose a neutral facilitator. The role of the facilitator is to guide the sprint.

- **Block five full days on the calendar**

Reserve time with your sprint team from 9 a.m. to 5 p.m. Monday through Friday. Make sure you include enough coffee breaks and a lunch break.

- **Book a room with two whiteboar**

Reserve a sprint room for the entire week. If it does not have two whiteboards in it already, buy some or improvise. Additionally, book a second room for the interviews scheduled on Friday.

- **Order healthy snacks for the week**

Providing nutritious snacks will help maintain the team's energy levels throughout the day. Offer wholesome foods such as apples, bananas, yogurt, cheese, and nuts. For an additional boost, include dark chocolate, coffee, and tea. Ensure there is an ample supply for all team members.

## Planner

### Monday, Month Day: Map, ask, and target

Monday’s structured discussions create a path for the sprint week and identify the problem. In the morning, a long-term goal will be set. Next, the challenge is mapped. In the afternoon, the experts are interviewed to share what they know. Finally, a target is picked to narrow down the scope of the sprint: an ambitious but manageable piece of the problem that can be progressed in one week.

| 8:00-9:00 | Room setup (Facilitator). |
| --- | --- |
| 09:00–09:30 | - - - - Welcome. - Introduction. - Participants introduction. |
| 09:30–10:25 | - - - - Set a long-term goal       - List sprint questions. |
| 10:25–10:35 | Morning break |
| 10:35–12:00 | - - - - Make a map: map each step of the workflow or patient journey. |
| 12:00–12:45 | Lunch break. |
| 13:00–14:45 | - Interview with experts: collect challenges during the interviews |
| 14:45–15:00 | Morning break |
| 15:00–15:30 | - Organize individual challenges in groups. - Vote on the most relevant challenges. |
| 15:30–16:00 | - Pick a target. |

**Tuesday, Month Day: Sketch**

After a full day of understanding the problem and choosing a target for the sprint, on Tuesday, the focus lies on solutions. The day starts with inspiration: a review of existing ideas to remix and improve. Then, in the afternoon, each person will sketch, following a four-step process that emphasizes critical thinking over artistry.

| 09:00–09:30 | - - - - Welcome. - Introduction. |
| --- | --- |
| 09:30–11:30 | - - - - Get inspired: search for concept inspiration. Think out of the box, the inspiration may come from completely different concepts or interfaces. |
| 11:30–12:00 | - - - - Present inspiration: everyone pitches the inspiration for their concept |
| 12:00–13:00 | Lunch break. |
| 13:00–16:00 | - The Four-Step Sketch: all team member individually sketch their concepts in four frames. |
| 16:00 | Good-bye. |

**Wednesday, Month Day: Decide**

By Wednesday morning, a collection of solutions will be available. While this is advantageous, it also presents a challenge. It is impractical to prototype and test all solutions; therefore, a single, robust plan is required. In the morning, each solution will be critically evaluated to determine which ones have the highest potential to achieve the long-term objective. In the afternoon, the selected scenes from the sketches will be integrated into a storyboard, forming a step-by-step plan for the prototype.

| 09:00–09:30 | - - - - Welcome. - Introduction. |
| --- | --- |
| 09:30–11:30 | - Pitch the concepts: each team member pitches his or her concept(s). |
| 11:30–12:00 | - Concept Decisions: decide on the best concept as a whole and on pieces of concepts. Use the winning concept as a base and adjust it with winning pieces of concepts from the other concepts. |
| 12:00–13:00 | Lunch break. |
| 13:00–16:00 | - Make a Storyboard: a storyboard illustrates the sequence of steps a user follows within the system. |
| 16:00 | Good-bye. |

**Thursday, Month Day: Prototype**

On Wednesday, a storyboard was created. On Thursday, a "fake it" approach will be adopted to transform that storyboard into a prototype. A realistic façade is sufficient for testing, and the advantage is that by concentrating on the customer-facing aspect of the product, the prototype can be completed in a single day. Additionally, on Thursday, preparations for Friday's test will be finalized by confirming the schedule, reviewing the prototype, and drafting an interview script.

| 09:00–09:30 | - - - - Welcome. - Introduction. |
| --- | --- |
| 09:30–12:00 | - Divide: divide tasks. Some team members will focus on generating or collecting realistic figures and images, others will focus on developing the interactive prototype. - Prototype |
| 12:00–13:00 | Lunch break. |
| 13:00–14:30 | - Prototype - Stitch it together. |
| 14:30–15:00 | - Trial run |
| 15:00–16:00 | - Finish the prototype |
| 16:00 | Good-bye. |

**Friday, Month Day: Test**

The sprint commenced with a significant challenge, an excellent team, and minimal resources. By Friday, promising solutions were developed, the best option was selected, and a realistic prototype was constructed. This alone constitutes a remarkably productive week. However, the process will be extended further by conducting customer interviews and gathering feedback on the prototype. This testing phase validates the entire sprint: by the end of the day, the extent of progress and the subsequent steps will be clearly understood.

| 09:00–09:30 | - - - - Welcome. - Introduction. |
| --- | --- |
| 09:30–12:00 | - Expert testing: present prototype in interviews and gather feedback. |
| 12:00–13:00 | Lunch break. |
| 13:00–17:00 | - Plan: define which features require finetuning. Plan which steps come after the design sprint and divide responsibilities. |
| 17:00 | Good-bye. |
