# Supplementary material for: Toward a human‐centric co‐design methodology for AI detection of differences between planned and delivered dose in radiotherapy
Source: J Appl Clin Med Phys. 2025 Mar 31;26(6):e70071. doi: 10.1002/acm2.70071 (PMC12148753; doi:10.1002/acm2.70071)
Supplement: Supplementary file 3 — Supporting information [file ACM2-26-e70071-s003.docx]

# Appendix III Expert user evaluation

Feedback on AI Certainty and Handling False Positives

1. Expert-user 1 emphasized the need for clarity on AI certainty levels and handling false positives. Suggested that understanding what different certainty percentages mean is crucial for trust.
2. Expert-user 2 highlighted the importance of AI certainty in decision-making. Suggested that high certainty would lead to acceptance without verification, while low certainty would require detailed review.
3. Expert-user 3 appreciated the AI certainty feature but noted that low-certainty cases should be highlighted (e.g., using color coding) to avoid being overlooked.

Visualization Preferences

1. Expert-user 1 stressed the importance of 3D visualization of the 3D dose in combination with anatomical information for better interpretation of errors. Preferred having detailed dose distributions and anatomical images.
2. Expert-user 2 suggested that 3D dose visualization is often easier for users and emphasized the need for dynamic, time-resolved information to understand treatment variations.
3. Expert-user 3 preferred having the 3D CT image with a heat map overlaid to understand the AI's analysis better. Found the current gamma map insufficient without anatomical context.

Integration and Accessibility

1. Expert-user 1 suggested integration into existing systems for a seamless workflow. Emphasized the need for easy access to notifications and comments during treatment sessions.
2. Expert-user 2 mentioned the importance of connectivity with existing systems and workflow engines. Suggested that the tool should be flexible enough to integrate with various dose verification methods.
3. Expert-user 3 considered the possibility of using the tool as a standalone system but acknowledged the need for integration with patient record systems for a connected workflow.

Trust and Reliability

1. Expert-user 1 highlighted the need for evidence of the AI's accuracy through tested examples to build trust. Rated trustworthiness lower due to the need for more reliability evidence.
2. Expert-user 2 expressed trust in the AI's potential but emphasized the need for reliable AI models. Suggested that trust would increase with demonstrated accuracy over time.
3. Expert-user 3 found the AI trustworthy but noted that continuous improvement through user feedback is essential. Rated trustworthiness moderately high but acknowledged the need for further validation.

Use in Clinical Practice

1. Expert-user 1 saw the tool as potentially very useful if it can run in the background and highlight only significant errors. Interested in using the tool for every fraction.
2. Expert-user 2 suggested that the tool could assist significantly in clinical practice by automating routine checks and highlighting critical issues. Emphasized the need for trend analysis over multiple fractions.
3. Expert-user 3 found the tool simple and easy to use, with the potential to save time and improve workflow. Suggested that the tool could act like a trained resident physicist, doing preliminary analysis for the user.

Ratings

- Usefulness: All rated the tool highly useful (6-7 out of 7).
- Ease of Use: Generally rated easy to very easy to use (5-7 out of 7).
- Safety: Seen as potentially increasing patient safety, but ratings varied (5-6 out of 7).
- Ease of Learning: Rated very easy to learn (7 out of 7).
- Trustworthiness: Rated moderately high but with room for improvement (4-6 out of 7).

Conclusion

While all three evaluations were positive and highlighted the tool's potential to improve clinical workflows, the main differences lie in their specific feedback on AI certainty, visualization preferences, integration needs, and trust-building measures. Each evaluator provided unique insights that can help refine and enhance the DGRT.AI tool.
